# Supplementary figures and images for: Structural Characterization of Foxtail Millet (Setaria italica) Polysaccharides and Evaluation of Its Antioxidant and Immunostimulatory Activities
Source: Antioxidants (Basel). 2025 Jan 20;14(1):113. doi: 10.3390/antiox14010113 (PMC11761630; doi:10.3390/antiox14010113)

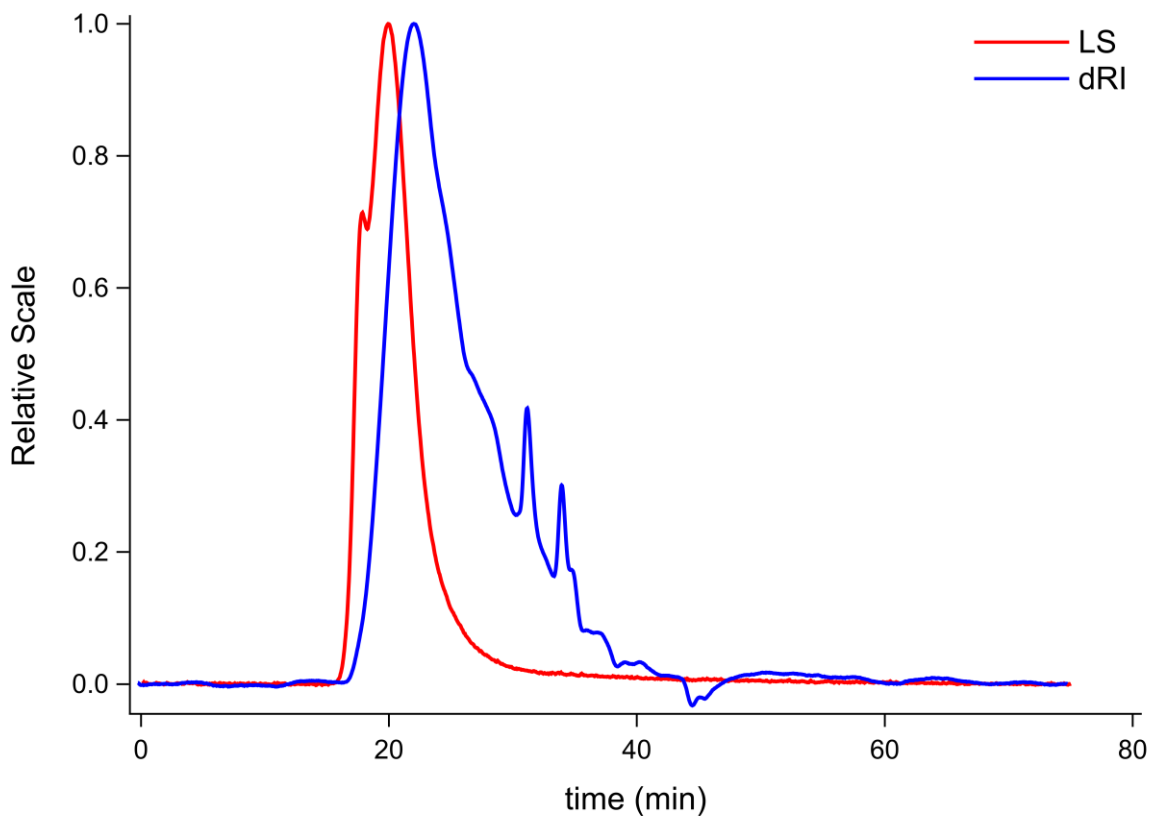

Supplement: Supplementary file 1 [file antioxidants-14-00113-s001.zip › Figure S1.pdf]

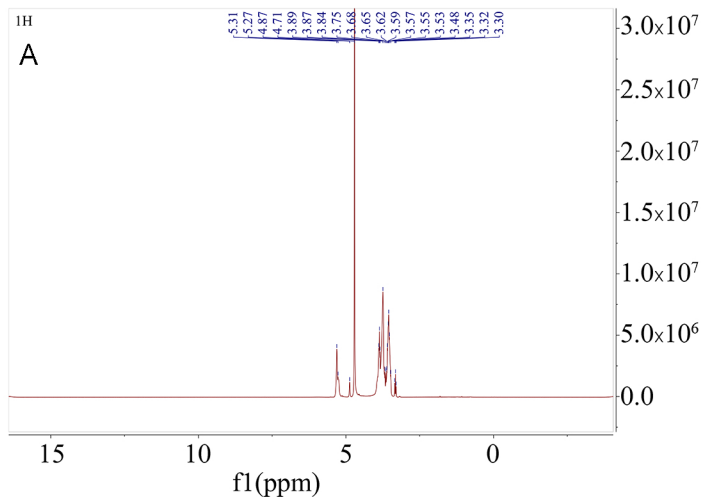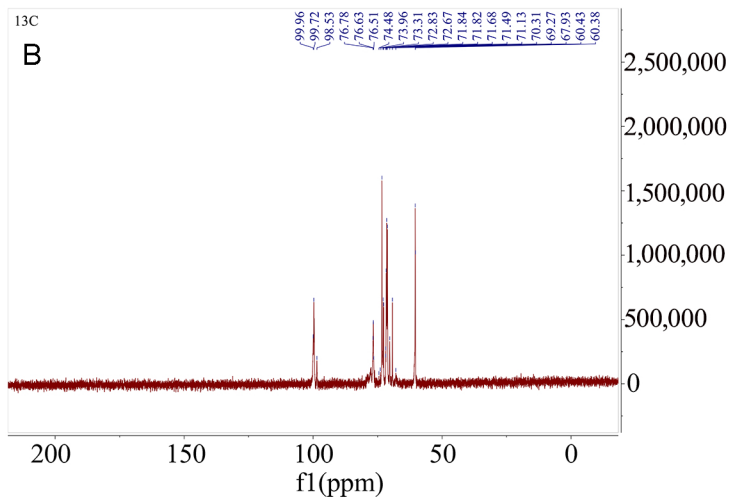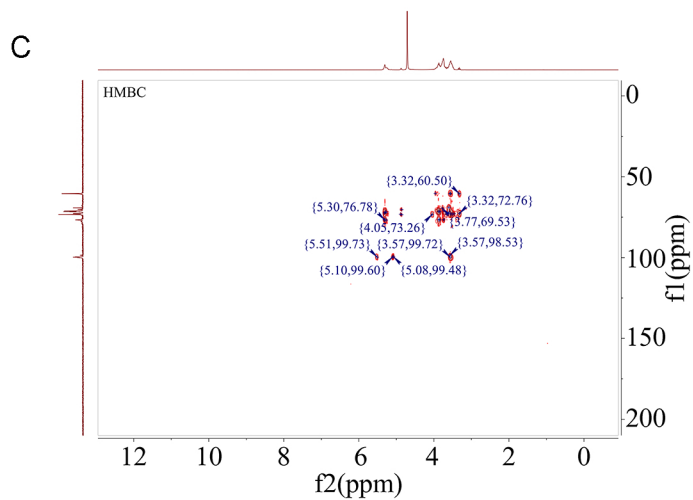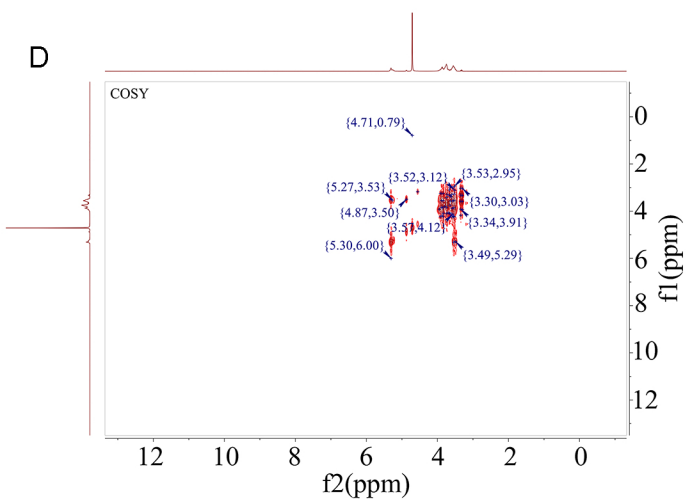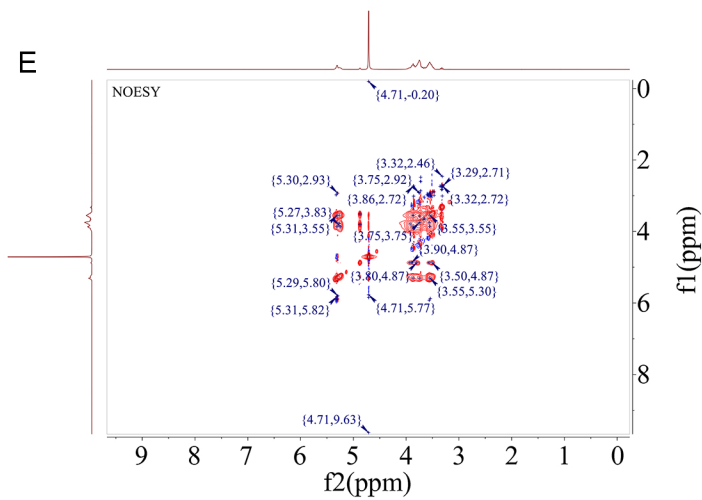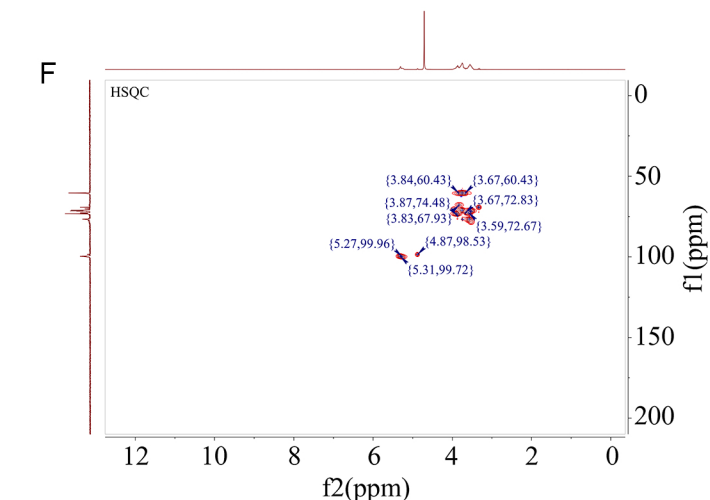

Supplement: Supplementary file 1 [file antioxidants-14-00113-s001.zip › Figure S2.pdf]
